# Oxidative stress in maternal milk and cord blood in gestational diabetes mellitus: a prospective study

**DOI:** 10.1590/1516-3180.2021.0209.R1.25082021

**Published:** 2022-03-18

**Authors:** Fırat Erdoğan, Evrim Şenkal, Ömer Faruk Özer, İlke Özahi İpek, Şükriye Leyla Altuntaş, Şükriye Özde

**Affiliations:** I MD. Associate Professor, Department of Pediatrics, School of Medicine, Istanbul Medeniyet University, Istanbul, Turkey.; II MD. Physician, Department of Pediatrics, School of Medicine, Istanbul Medipol University, Istanbul, Turkey.; III MD. Physician, Department of Biochemistry, School of Medicine, Bezmiâlem Foundation University, Istanbul, Turkey.; IV MD. Professor, Department of Pediatrics, School of Medicine, Istanbul Medipol University, Istanbul, Turkey.; V MD. Physician, Department of Obstetrics and Gynecology, School of Medicine, Istanbul Medipol University, Istanbul, Turkey.; VI MD. Physician, Department of Pediatrics, School of Medicine, Düzce University, Düzce, Turkey.

**Keywords:** Diabetes mellitus, gestational, Stress, oxidative, Antioxidants, Gestational diabetes mellitus, Oxidative stress index, Total antioxidant capacity, Total oxidant status

## Abstract

**BACKGROUND:**

Reduced antioxidant defenses may reflect a poor protective response against oxidative stress and this may be implicated in progression of gestational diabetes mellitus (GDM). Oxidative stress induced by hyperglycemia plays a major role in micro and macrovascular complications, which imply endothelial dysfunction.

**OBJECTIVE:**

Our aim in this study was to investigate the association between GDM and oxidative stress markers measured in plasma, with regard to revealing changes to total antioxidant capacity (TAC) and total oxidant status (TOS) among mothers showing impairments in oral glucose tolerance tests (OGTTs).

**DESIGN AND SETTING:**

Prospective study at a university hospital in Turkey.

**METHODS:**

The study group consisted of 50 mothers with GDM, and 59 healthy mothers served as controls. Umbilical cord blood samples were taken from all mothers during delivery and breast milk samples on the fifth day after delivery. TAC, TOS, thiol and disulfide levels were measured.

**RESULTS:**

No statistically significant relationship between the blood and milk samples could be found. An analysis on correlations between TAC, TOS and certain parameters revealed that there were negative correlations between TOS and total thiol (r = -0.386; P < 0.001) and between TOS and disulfide (r = -0.388; P < 0.001) in milk in the control group. However, these findings were not observed in the study group.

**CONCLUSION:**

Our findings suggested that a compensatory mechanism of oxidative stress was expected to be present in gestational diabetes mellitus and that this might be ameliorated through good glycemic regulation and antioxidant supplementation.

## INTRODUCTION

There is a balance between the reactive oxygen compounds produced by different mechanisms and the antioxidant systems generated by the enzymatic and non-enzymatic processes that neutralize these oxygen compounds.^1^ The term “oxidative stress” expresses the imbalance between oxidant/antioxidant molecules in favor of oxidants that cause aging and diseases.^2,3^ Oxidative stress has also been implicated in the pathogenesis of vascular diseases, such as atherosclerosis, diabetes and hypertension, which result from an imbalance between increased formation of reactive oxygen species (ROS) and synthesis of anti-oxidative defense mechanisms.

Under diabetic conditions, the end products of abnormal glucose metabolism lead to increased synthesis of ROS. Formation of advanced glycation end products, activation of hexosamine biosynthetic pathway, increased lipid peroxidation and an impaired antioxidant defense system result in accumulation of free radicals, eventually.^5^ Experimental studies have revealed increased levels of free oxygen radicals in diabetic pregnancy.^6^ Furthermore, it was observed in an animal study that antioxidant supplementation can decrease occurrence of malformations in offspring.^7^ Both experimental and clinical studies have shown that in the presence of gestational diabetes, there is enhanced oxidative stress, which is detectable in maternal and neonatal blood samples, placental tissue and amniotic fluid.^5^

It has been suggested that oxidative stress plays a role in maternal and fetal complications of diabetic pregnancies.^8–10^ Pregnancy alone may represent an oxidative stress condition.^11^ Associations between gestational diabetes mellitus (GDM) and oxidative stress markers measured in plasma have already been reported, with limited consensus. This suggests that oxidative stress may be implicated in GDM progression and/or pathogenesis and that reduced antioxidant defenses may reflect a poor protective response against oxidative stress.^9,12,13^

Oxidant agents may be produced either endogenously or exogenously, as in the case of ultraviolet rays, active smoking or passive exposure to cigarettes.^14^ Although oxidant agents can be measured one-by-one, it is widely preferred to measure total oxidant status (TOS) because the individual measurement approach involves increased work time and complicated techniques for each agent.^15^ Similarly, antioxidants can be measured through the total antioxidant capacity (TAC) rather than one-by-one.

When a balance between TAC and TOS is needed, there may be a shift in favor of TOS; this is called oxidative stress (OS). One of the most important reasons for OS is impaired glucose tolerance, which manifests as obvious diabetes mellitus (DM) or through impairment seen in an oral glucose tolerance test (OGTT) during pregnancy. Previous studies revealed that the levels of oxidant molecules increase in diabetic animals, while antioxidants have been found to be decreased in diabetic patients.^16,17^

Oxidative stress induced through hyperglycemia plays a major role in micro and macrovascular complications, which imply endothelial dysfunction. Appropriate glycemic control prevents complications related to increased OS during pregnancy.^18^

## OBJECTIVE

Our aim in this study was to investigate GDM-related OS markers in maternal milk and cord blood, and to increase awareness of possible measures to be taken, through revealing the changes in TAC and TOS and in the OS index levels of mothers with impairments seen in OGTTs.

## METHODS

Pregnant women were routinely assessed by means of a 50 g glucose loading test at gestational ages of between 24 and 28 weeks. Those whose blood glucose levels were < 140 mg/dl were accepted as normal and were included in the control group. Those whose blood glucose levels were ≥ 140 mg/dl were given a 100 g glucose loading test. In accordance with the suggestions of the American Diabetes Association (ADA), patients with high glucose levels in two out of four tests conducted at hours 0, 1, 2 and 3 were diagnosed as having GDM and were included in the study group.

The patients in the study group attended consultations at the department of endocrinology in order to regulate their blood glucose levels. Their hemoglobin A1c (HbA1c) levels were measured, and their blood glucose levels were monitored. Out of the 50 patients, the blood glucose levels of 47 of them were regulated only through dietary control. One patient took oral antidiabetic medication, and two patients needed insulin treatment for appropriate glucose regulation. The exclusion criteria of this study were situations of histories of chronic disease, histories of infection during pregnancy, smoking during pregnancy, substance abuse history, pregnancy at ages < 18 years or > 35 years, consanguineous marriage or previous histories of complications of pregnancy or delivery.

The participants were asked about medication or food supplementation use that could have affected their antioxidant capacity (gingko, guelder-rose, vitamin C, vitamin E, coenzyme Q-10, resveratrol, lipoic acid, etc.).

Informed parental consent was obtained for the cord blood collection and the study was approved by the Ethics Committee of the School of Medicine of Istanbul Medipol University, in Istanbul, Turkey (approved on January 23, 2015; number 23.01.2015/10840098-06).

After delivery of the baby, the umbilical cord was clamped and cut. Cord blood was drawn from the umbilical vein within two minutes of delivery. In order to standardize breast milk sample collection, these samples were collected from all mothers on the fifth day. Serum samples collected from the study and control groups were immediately separated from the cells through centrifugation at 3,000 g for 10 minutes. They were then stored at −80 °C until further analysis of the native thiol, total thiol, disulfide, TOS, TAC and OSI.

### Measurement of total oxidant status

Serum TOS values were measured through an assay based on an automated measurement method developed by Erel.^15^ Oxidants present in the sample oxidize ferrous ions to ferric ions. Ferric ions are manifested through a colored complex with xylenol orange in an acidic medium. The color intensity, which can be assessed spectrophotometrically, is dependent on the total quantity of oxidant molecules present in the sample. TOS values are expressed in terms of micromolar hydrogen peroxide (H_2_O_2_) equivalents per liter (μmol H_2_O_2_ eq/l). Serum thiol and disulfide levels are expressed as micromoles per liter (μmol/l).

### Measurement of total antioxidant capacity

The total antioxidant capacity (TAC) of the serum samples was assayed using a method developed by Erel.^19^ In this method, the characteristic blue color of the 2, 2’-azino-bis (3-ethyl benzothiazoline-6-sulfonic acid) (ABTS) cation is converted back to its neutral form by any antioxidant present in the sample. This reaction is also monitored spectrophotometrically. The assay results are expressed in mmol Trolox equivalent per liter.

### Measurement of plasma oxidative stress index

There are several indexes for measuring OS in humans and most of them make it possible to diagnose and differentiate OS related to human health and disease. The oxidative stress index (OSI) is one of these indexes, and this has been proven to be reliable and practical.^20^

The OSI was calculated using the following formula: OSI (arbitrary unit) = TOS (μmol H_2_O_2_ eq/l)/TAC (mmol Trolox eq/l) x 100 (to represent a percentage ratio).

For the analysis of the present study, in addition to descriptive statistical methods (i.e. frequency, percentage, mean and standard deviation), the Kolmogorov-Smirnov test was used to assess whether the data showed normal distribution. Independent-sample t tests were applied to compare quantitative data between pairs of groups. Pearson correlation analysis was used to evaluate relationships between the groups’ quantitative data. The results were evaluated at a 95% confidence interval, and P < 0.005 was considered to be significant in the two-way analyses.

## RESULTS

A total of 109 participants with a mean age of 28.8 ± 7.2 years were included in our study. There were 50 patients with GDM, with a mean age of 25.2 ± 6.4 years. The healthy control group included 59 patients, with a mean age of 27.6 ± 7.6 years. The normal spontaneous delivery rate was 12% (n = 6) in the study group and 11.8% (n = 7) in the control group. There were no significant differences with regard to age, body mass index (BMI) or obstetric history between the groups (for all parameters, P > 0.05). Two of the pregnant women with GDM required insulin treatment, and one patient needed to take oral antidiabetics. For 47 patients, their blood glucose levels were regulated through dietary control. The mean HbA1c level of the patients was 5.2% ± 0.4%.

**Figure 1.**
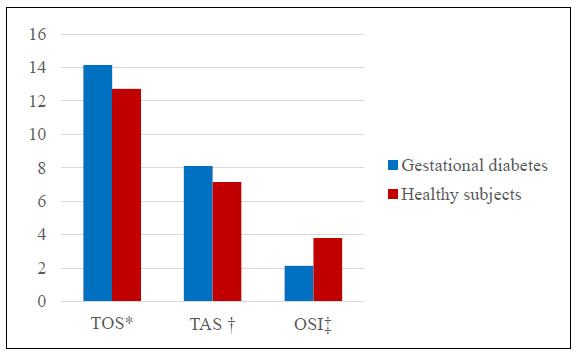
Comparison of blood total oxidant status (TOS) *(μmol H_2_O_2_ eq/l), total antioxidant capacity (TAS) ^†^(mmol Trolox eq/l) and oxidative stress index (OSI) ^‡^(CarrU/(mmol HClO/ml) levels between women with gestational diabetes mellitus and healthy pregnant women.

**Figure 2.**
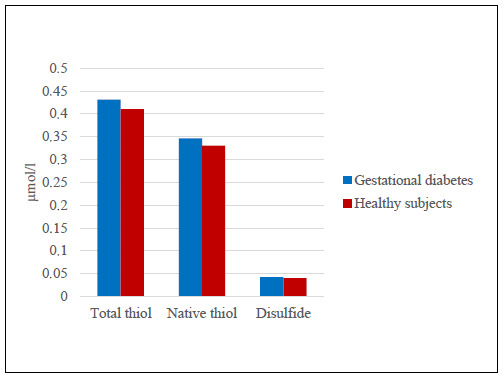
Comparison of blood thiol total, thiol native and disulfide levels in women with gestational diabetes mellitus and healthy pregnant women.

**Figure 3.**
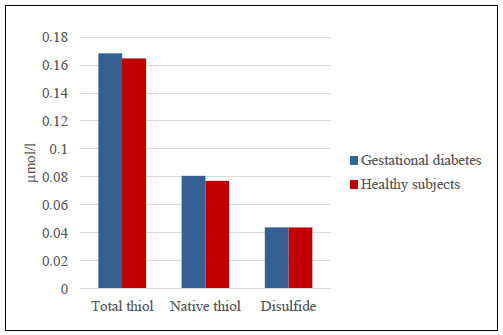
Comparison of milk thiol total, thiol native and disulfide levels in women with gestational diabetes mellitus and healthy pregnant women.

**Figure 4.**
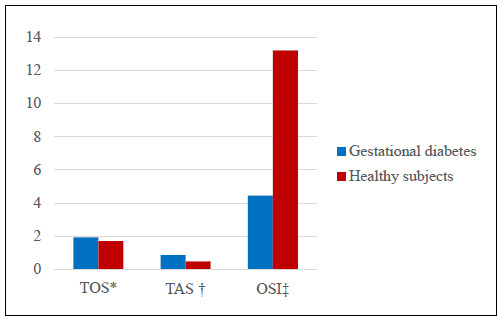
Comparison of milk total oxidant status (TOS) *(μmol H_2_O_2_ eq/l), total antioxidant capacity (TAS) ^†^(mmol Trolox eq/l) and oxidative stress index (OSI) ^‡^(CarrU/mmol HClO/ml) levels in women with gestational diabetes mellitus and healthy pregnant women.

The values for laboratory findings (TAC, TOS, OSI, disulfide, total thiol and native thiol) in the groups are presented in Table 1. No statistically significant relationship between blood and milk samples could be found. Although the TAS-milk, milk total thiol, milk N-thiol and milk disulfide levels in the study group were higher than those of the healthy group, there were no statistically significant differences between the groups (P > 0.05) (Figures 2 and 3). The OSI level in milk was lower in the study group than in the control group but the OSI in blood was higher (P > 0.05) (Figures 1 and 4). An analysis on the correlation between TAC, TOS and certain parameters in the control group revealed that there were negative correlations between TOS and total thiol (r = -0.386; P < 0.001) and between TOS and disulfide (r = -0.388; P < 0.001) in milk. However, these findings were not seen in the study group (Table 2 [control group] and Table 3 [study group]).

**Table 1.** Mean scores of oxidant and antioxidant levels of women with gestational DM and healthy pregnant women

	Gestational diabetes mellitus group Mean (SD)	Control group Mean (SD)	P
**Antioxidant levels**			
Blood total thiol (μmol/l)	0.409 (0.05)	0.425 (0.055)	> 0.5
Milk total thiol (μmol/l)	0.167 (0.02)	0.160 (0.018)	
Blood N-thiol (μmol/l)	0.326 (0.05)	0.343 (0.055)	
Milk N-thiol (μmol/l)	0.078 (0.02)	0.075 (0.019)	
**Oxidant levels**			
Blood disulfide (μmol/l)	0.041 (0.003)	0.041 (0.003)	> 0.5
Milk disulfide (μmol/l)	0.044 (0.004)	0.042 (0.005)	
**Total oxidant status**			
Blood (μmol H_2_O_2_/l)	8.288 (4.35)	9.792 (5.08)	> 0.5
Milk (μmol H_2_O_2_/l)	2.025 (0.94)	2.052 (0.82)	
**Total antioxidant capacity**			
Blood (mmol Trolox eq/l)	4.455 (3.43)	5.572 (4.25)	> 0.5
Milk (mmol Trolox eq/l)	0.756 (0.79)	0.616 (0.47)	
**Oxidative stress index**			
Blood	48.69 (113.31)	38.30 (44.52)	> 0.5
Milk	54.92 (50.21)	92.72(139.58)	

SD = standard deviation.

**Table 2.** Correlation analysis on TAC, TOS, OSI, total thiol, N-thiol and disulfide in healthy pregnant women

Oxidant and antioxidant levels in blood and milk	Blood TOS	Blood TAC	Blood total thiol	Blood N-thiol	Blood disulfide	Milk TOS	Milk TAC	Milk total thiol	Milk N-thiol
**Blood TOS**	**1**								
**Blood TAC**	0.983^**^								
**Blood total thiol**	0.345	0.277							
**Blood N-thiol**	0.254	0.184	0.992^**^						
**Blood disulfide**	0.467*	0.471*	−0.035	−0.159					
**Milk TOS**	0.329	0.342	−0.084	−0.121	0.301				
**Milk TAC**	0.105	0.018	0.132	0.092	0.308	0.294*			
**Milk total thiol**	−0.381	−0.405	−0.119	−0.085	−0.262	−0.386^**^	−0.156		
**Milk N-thiol**	−0.,374	−0.321	−0.,248	−0.235	−0.087	−0.146	−0.119	0.807^**^	
**Milk disulfide**	0.054	−0.048	0.220	0.252	−0.270	−0.388^**^	−0.052	0.276*	0.345^**^

*P < 0.5; **P < 0.01. TOS = total oxidant status; TAC = total antioxidant capacity; OSI = oxidative stress index.

**Table 3.** Correlation analysis on TAC, TOS, OSI, total thiol, N-thiol and disulfide in women with gestational diabetes mellitus

Oxidant and antioxidant levels in blood and milk	Blood TOS	Blood TAC	Blood total thiol	Blood N-thiol	Blood disulfide	Milk TOS	Milk TAC	Milk total thiol	Milk N-thiol
**Blood TOS**	1								
**Blood TAS**	0.983**								
**Blood total thiol**	0.462*	0.414*							
**Blood N-thiol**	0.456*	0.401*	0.993**						
**Blood disulfide**	−0.207	−0.115	0.257	0.139					
**Milk TOS**	0.123	0.155	−0.047	−0.065	0.123				
**Milk TAC**	−0.120	−0.037	−0.222	−0.257	0.222	0.081			
**Milk total thiol**	−0.044	−0.032	0.229	0.246	−0.077	−0.013	−0.021		
**Milk N-thiol**	−0.067	−0.048	0.271	0.294	−0.107	0.126	−0.042	0.934**	
**Milk disulfide**	0.067	0.044	−0.207	−0.232	0.137	−0.326	0.050	0.429**	0.080

*P < 0.5; **P < 0.01. TOS = total oxidant status; TAC = total antioxidant capacity; OSI = oxidative stress index.

The detailed histories of the patients revealed that 94% of them had been taking preparations that included multivitamins (n = 48), 15% had been taking medications that included omega-3 (n = 8) and 15% had been consuming foodstuffs that had antioxidant properties (although the levels and contents of these antioxidant molecules were not well known).

## DISCUSSION

Studies investigating the association between gestational diabetes mellitus (GDM) and oxidative stress have reported inconsistent findings. Although there is no irrefutable proof of a relationship between GDM and oxidative stress, it has been shown that the levels of molecules that give rise to OS are influenced by GDM.^4,21^ In spite of evidence that oxidative stress plays an important role in the pathogenesis of DM, there is debate on its role in GDM and the impact of the oxidant/antioxidant balance.^11^

Although the parameters in both cord blood and maternal milk samples in the gestational diabetes group of our study were than those of the healthy group, there were no statistically significant differences between the groups in our study. In addition, the OSI did not differ. On the other hand, the milk TOS level was negatively correlated with the milk thiol level in the non-GDM pregnant group, whereas increased milk TAC occurred as a compensation. This finding was interpreted as a sign that this compensation condition was impaired in GDM.

Oxidative stress impacts mortality and morbidity in all age groups. This may lead to disturbances during pregnancy and the postnatal period. One of the factors increasing the OSI during pregnancy is impaired glucose tolerance.^18^ Reactive oxygen species (ROS) and nitrogen production can alter several cellular components, as well as the redox state. All of these are maintained by complex mechanisms that lead to insulin resistance, b-cell dysfunction, glucose intolerance and type 2 DM (T2DM).^22^ Animal experiments have shown that hyperglycemia increases the oxidative damage of deoxyribonucleic acid (DNA) and plays a role in the pathogenesis of DM complications.^18,23^ It has also been shown that TAC, TOS and OSI were significantly increased in the cord blood of infants of diabetic mothers, compared with healthy controls.^24^ Several studies have reported occurrences of impaired antioxidant/oxidant balance in GDM caused by increased levels of reactive oxygen species, such as protein glycation, glucose oxidation and lipid peroxidation.^6,9,10^

Fluctuations in TAC and TOS levels can both result from and cause hyperglycemia. Decreased TAC levels in GDM patients increase the amount of insulin required for adequate glycemic control.^25^ However, findings regarding the role of the antioxidant system in this imbalance have been conflicting. Some of the studies reported decreased maternal TAC levels in GDM but others did not.^8–10^ The presence of oxidant stress in GDM was explained by Biri et al.^6^ in terms of impaired antioxidant defense mechanism and increased free radical production. This result means that there is a compensatory increase in the activity of antioxidant system, to cope with the elevated free radical production.

Concerning the possible molecular mechanisms leading to oxidant stress in GDM, the result have, however, been divergent. For instance, Sarıkabadayı et al. suggested that impaired glycemic control is responsible for elevated oxidative activity in infants of diabetic mothers rather than decreased antioxidant enzyme defense systems.^8^ However, since OSI was still higher than in the control group, this elevation in TAC was not enough to establish an impaired oxidant-antioxidant balance.^26^ Some researchers have argued that the increase in oxidative activity in GDM is not secondary to a deficiency in the antioxidant defense, but is due to impaired glycemic control.^26^ It has been suggested that oxidative stress, which mainly arises from hyperglycemia, is implicated in the development of diabetic complications. Moreover, impairment of the antioxidant system may also play a role in occurrences of oxidant stress in GDM.^6^

Studies have found that mothers experience increased OS and inflammatory responses during late gestation and lactation. These symptoms are likely to affect not only the wellbeing of the mothers, but also the health of their offspring.^27^ Impairment of the balance of oxidants and antioxidants can also affect newborns, because this period of development is a more sensitive phase with low antioxidant capacity.^28^ In our study, neither the cord blood of infants of diabetic mothers nor the milk TAC, TOS or OSI levels were significantly different between the groups. In a similar study, the TAC levels were similar, and the result was interpreted as a compensatory response (24). Human milk contains many bioactive antioxidant compounds that are part of the body’s defense system against the actions of various free radicals, such as superoxide dismutase, glutathione peroxidase, vitamins C, A and E and α-carotenes.^29^ Moreover, in diabetic animal models, antioxidant therapy has been shown to be effective for alleviating the deleterious effects of GDM on the fetus.^30^ It is still unclear whether a diet rich in antioxidants, or antioxidant supplementation of the diet, might improve oxidative stress in GDM.^11^

We could not find any relationship between the TAC, TOS and OSI levels of the study and control groups, and we have two explanations for this result. Firstly, as the mean HbA1c level was 5% ± 0.4%, we can state that blood glucose levels were regulated adequately and that these results were reached under conditions of good glycemic regulation in the study group. Additionally, consumption of antioxidant foods was not taken into account, and this was a limitation of our study that may have affected the results. Moreover, antioxidants can be used to decrease OSI, besides decreasing TOS. For example, omega-3 fatty acids have been shown to decrease malondialdehyde (MDA) levels in GDM patients.^31^ In the literature, it was shown that when lipid peroxidation products (MDA) increased, levels of glutathione peroxidase (GPX) and superoxide dismutase were decreased in pregnant women with GDM.^9,32^ However, Orhan et al. found significantly increased erythrocyte selenium (Se)-GPX activity in insulin-dependent diabetic pregnancy.^12^ An animal study by Kemse et al. revealed that supplementation of micronutrients such as folic acid, B12 and omega-3 fatty acids can decrease OS and inflammation related to preeclampsia.^33^ Use of supplementation of antioxidant molecules was reported by Gurkan et al., in situations of high MDA and low selenium levels in infants with acute bronchiolitis.^34^ In another animal study, resveratrol, a plant-derived antioxidant, was reported to prevent embryopathy resulting from exposure to high glucose levels and increased OSIs.^35^

Among our subjects, 94% of our study group declared that they had been taking some medications. This may have influenced our results.

Moreover, many mothers significantly modify their diets during pregnancy and lactation: in particular, they demand products that are safe and free from synthetic additives. To meet consumer needs, the food industry has begun to use natural antioxidant extracts as food preservatives.^36^ Thus, another limitation of our study was that we did not record the participants’ food consumption: these foods might have had oxidant/antioxidant effects.

## CONCLUSION

Impaired glucose tolerance, one of the most common problems experienced during pregnancy, can produce many complications in newborns through increasing oxidative stress. Thus, in this study, we sought to emphasize the importance of proper glucose regulation for preventing health problems among newborns. Our findings suggested that a compensatory mechanism of oxidative stress was expected to be present in gestational diabetes mellitus and that this might be ameliorated through good glycemic regulation and antioxidant supplementation.
